# Possibility of reconstruction of dental plaster cast from 3D digital study models

**DOI:** 10.1186/1475-925X-12-49

**Published:** 2013-05-31

**Authors:** Magdalena Kasparova, Lucie Grafova, Petr Dvorak, Tatjana Dostalova, Ales Prochazka, Hana Eliasova, Josef Prusa, Soroush Kakawand

**Affiliations:** 1 Department of Stomatology, 2nd Medical Faculty, Charles University Prague, V Uvalu 84, 150 06, Prague 5, Czech Republic; 2 Faculty of Chemical Engineering, Department of Computing and Control Engineering, Institute of Chemical Technology, Technicka 5, 166 28, Prague 6, Czech Republic; 3 InstPrusa Research s.r.o., Senovazna 996/6, 110 00, Prague, Czech Republic; 4 Institute of Criminalistics, Prague, P.O. BOX 62/KUPStrojnicka 27, 17089, Prague, Czech Republic

**Keywords:** 3D print, Rapid prototyping, Plaster cast, Dentistry

## Abstract

**Objectives:**

To compare traditional plaster casts, digital models and 3D printed copies of dental plaster casts based on various criteria. To determine whether 3D printed copies obtained using open source system RepRap can replace traditional plaster casts in dental practice. To compare and contrast the qualities of two possible 3D printing options – open source system RepRap and commercially available 3D printing.

**Design and settings:**

A method comparison study on 10 dental plaster casts from the Orthodontic department, Department of Stomatology, 2nd medical Faulty, Charles University Prague, Czech Republic.

**Material and methods:**

Each of 10 plaster casts were scanned by inEos Blue scanner and the printed on 3D printer RepRap [10 models] and ProJet HD3000 3D printer [1 model]. Linear measurements between selected points on the dental arches of upper and lower jaws on plaster casts and its 3D copy were recorded and statistically analyzed.

**Results:**

3D printed copies have many advantages over traditional plaster casts. The precision and accuracy of the RepRap 3D printed copies of plaster casts were confirmed based on the statistical analysis. Although the commercially available 3D printing enables to print more details than the RepRap system, it is expensive and for the purpose of clinical use can be replaced by the cheaper prints obtained from RepRap printed copies.

**Conclusions:**

Scanning of the traditional plaster casts to obtain a digital model offers a pragmatic approach. The scans can subsequently be used as a template to print the plaster casts as required. Using 3D printers can replace traditional plaster casts primarily due to their accuracy and price.

## Background

Plaster casts offer the gold standard for monitoring and documenting the progression of treatment alongside photographs and imaging technologies [1]. It has been evident from experience that plaster casts present precise and reliable information about patients’ dental arches, position of teeth and their dimensions. Patients’ orthodontic data is usually obtained from measurements of dental casts by a digital caliper and are further stored for future use. Currently it is necessary to store the patients’ plaster casts for legislative and further clinical workups, however with the use of stored patients’ data in the digital form we not only retain the possibility of reproducing the dental casts when the necessities arise we also eliminate the unnecessary storage of plaster casts which are not readily required.

The disadvantage of using the plaster casts include the burden of their storage, risk of damage or breakage, their heavy weight and difficulties in sharing their data with other professionals involved in the patients’ care [2,3].

Digital technology has integrated into all branches of dental practice such as prosthodontics, conservative dentistry and orthodontics. These technologies include cone beam tomography, three dimensional facial imaging and various scanning techniques [4,5]. Many studies have proved that 3D digital models of dental casts have the same accuracy and precision as measurements made on traditional plaster casts [6-10] all this is achieved without significantly compromising the reliability of occlusal information [11]. Leifert et al. devoted their work comparing digital models and plaster casts. Their results are acceptable for clinical practice with the mean difference of less than 0.5 mm between measurements on digital models and plaster casts [12]. Digital study models were confirmed to be a valid alternative to traditional plaster casts in treatment planning for Class II malocclusion patients by Whetten et al. [13]. This study compared the reliability of decision making from data obtained from both the digital and traditional plaster casts models, which is an important step from theory to practice.

Here it must be mentioned that digitalization of patients’ data is susceptible to misuse as well as being prone to technical errors. These include limitations in storing large amounts of data due to storage capacity constraints of hardware, data loss and unauthorized access to the patients’ data. However most of these technical difficulties can be overcome by specialists’ input in their respective field. The available methods of digitalization include: plaster casts scanning by scanners (tactile or non-tactile), photo-optical digitalization (eg. stereophotogrammetry), computer tomographic methods and laser-optical methods.

Thus far only a single study has demonstrated the possibility of obtaining 3D object from digital data in dentistry. In the study carried out by Keating at al. a standard stereolithography (clear resin, 0,15mm layers) was employed to obtain 3D model of a scanned cast. However it was subsequently found that this study lacked sufficient accuracy as only two plaster casts were printed in 3D and used for all the measurements. The same study confirmed hand held digital calipers are reliable for cast measurements [14].

There are a number of available printers with the ability to print various 3D objects using different technologies. The most commonly used printers are FDM (fusion deposition modeling) where thin plastic line is laid down and builds up plastic object. RepRap machines are one of the available printers utilizing this technique. Powder based printers such as Zcorp (gypsum like powder glued by inkjet) or SLS (selective laser sintering) where nylon or similar type of thermoplastic powder is locally melted with laser beam). SLA (stereolitography) is another type of printer where UV curable resin is cured in desired shape by light source.

Despite providing the highest quality of printing, SLA remains relatively an expensive and slow option where a print can take up to 12 hours. By comparison to SLA printers, FDM printers offer poor quality prints with distinguishable layered surface. Open source RepRap printers, however, offer affordable prints which can provide both a close enough match in under an hour or highly detailed prints in few hours as required.

### Aim of the present study

1. To determine the possibilities of reversing 3D digital models obtained by scanning traditional plaster cast, into physical 3D shapes.

2. To elucidate whether the distance measurements on the plaster models and on the RepRap 3D printed copies are equivalent and comparable.

3. To determine whether RepRap 3D printed model can replace the process of plaster casts making and its potential benefits and advantages.

## Materials and methods

10 plaster casts used in this study were randomly selected and obtained from Orthodontic clinic of the Department of Stomatology, 2nd medical Faculty, Charles University, Prague. Informed consents were obtained from all patients or their legal representatives. No ethical approval was required for this study.

All plaster casts were poured from patient’s impressions (alginate impression material, Ypeen, Czech Republic) taken during orthodontic treatment. All of them were cast in conventional material (gypsum) and conventionally trimmed. There were no personal data written on the casts. They were subsequently numbered from 1 to 10. All of the plaster casts completely reproduced full arches, with no surface damage, loss of tooth material or breakage. The plaster casts were neither magnified nor landmarks premarked. Various orthodontic anomalies and positions of teeth were demonstrated to be taken as representative of commonly encountered orthodontists’ cases.

To capture 3D digital model of all plaster casts, inEos Blue scanner (Sirona, Austria) was used with the same protocol for all samples. InEos Blue is a non tactile scanner based on short-wavelength blue light. All plaster casts were scanned in multiple planes depending on the shape of the teeth and especially hard palate to cover all surfaces of the cast. Scanned data were saved as STL files; computer software inLab Biogeneric (Sirona, Austria) merged obtained data and composed a 3D digital model. The models were exported into the standardized stereolitography (STL) file format. The obtained 3D models of the plaster casts consist of approximately 260 000 vertices and 500 000 faces, and thus their size is approximately 25 MB. The resolution of the scanning (and of the obtained model too) is 19 μm, but the resolution of the RepRap printer is approximately 200 μm. Therefore we reduced and remeshed the models to approximately 55 000 faces using the open source mesh processing system MeshLab (Visual Computing Lab – ISTI – CNR, Italy) and Netfabb Studio Basic (netfabb GmbH, Germany). We used the Quadric edge collapse decimation method for reduction. The size of the reduced model is approximately 3 MB. Then the self-intersecting or duplicated faces, non-manifold edges and vertices were removed where needed. The last step of the STL model processing was filling of the holes which rendered the model ready and fully valid for the RepRap printer. Finally, the STL model was sliced into individual layers, the path of printing nozzle was computed and the STL was converted to GCODE file by Slic3r (Alessandro Ranellucci, Italy). The GCODE file contains all information needed by the 3D printer.

**Figure 1:**
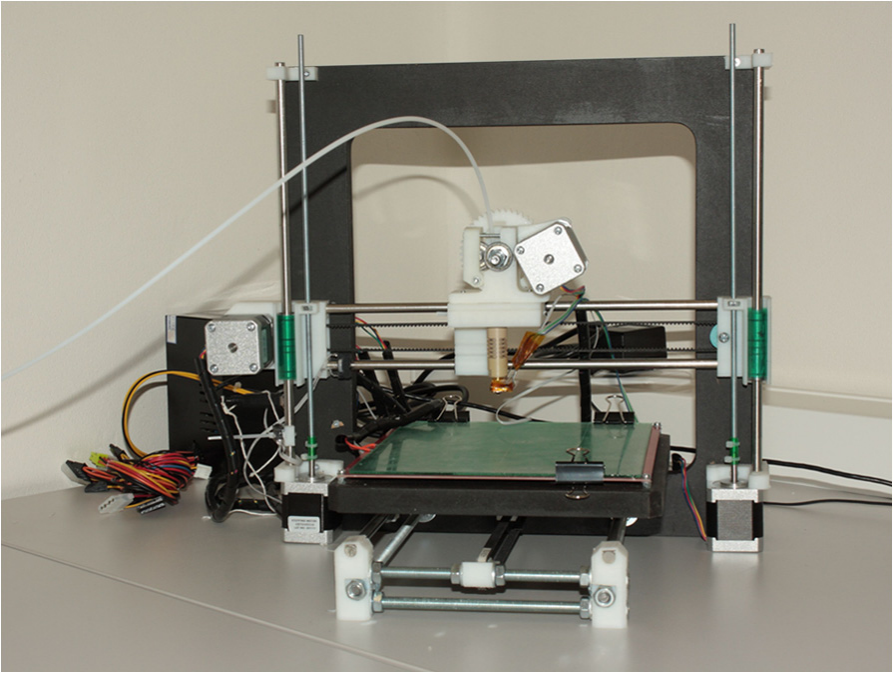
RepRap 3D printer.

Ten of the plaster casts were 3D printed on RepRap (Czech Republic) 3D printer (ABS plastic material, layer 0.35 mm, blue color) (Figure 1). Layer of 0.35 mm is the thinnest layer which can be achieved by RepRap printers at the moment. One of the models was also printed on ProJetHD3000 3D printer (3D Systems, USA, clear resin) to compare the distances and quality of models printed on open source and commercial printers.

**Figure 2:**
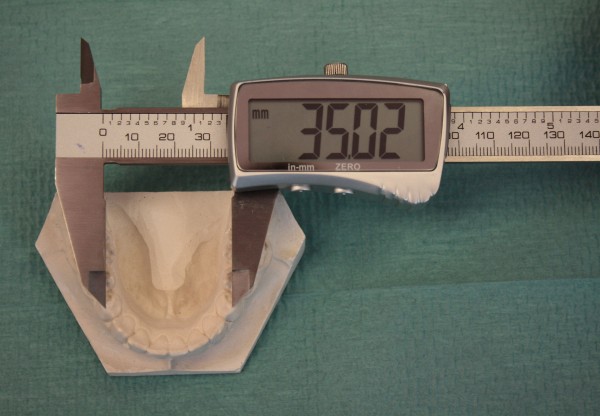
Hand held digital caliper with accuracy of 0.01mm used for measuring distances of specified points.

Similar linear measurements were taken plaster casts and 3D printed copies by a single trained evaluator after initial training. A few outlier measurements were not included in the study to improve the methodology of measurements by the digital caliper, all measurements were performed twice, with one week interval using hand held digital caliper (Festa, China, accuracy 0.01 mm) (Figure 2). A total of 160 measurements were statistically evaluated.

Measured dimensions were selected as the distance between:

x-plane: Intercanine distance – distance between the occlusal tips of canines

y-plane: The distance of tips of the canine and, mesio-palatal cusp of the first permanent molar in the jaw

z-plane: The clinical crown height of canine

Mixed distance: Distance between mesial edge of the first incisor and cusp of canine

### Statistical evaluation

All Data obtained and presented here were subjected to statistical evaluation.

Reproducibility of the measurements on the plaster models and RepRap 3D printed copies were tested by paired t- test [15] (Table 1). Here a necessary assumption has been made that the differences between repeated measurements are from a normal distribution. The assumption that the measurements belong to a normal distribution, was subsequently confirmed using the Lilliefors test [16-18].

**Table 1:** **Results of paired *t*-test of the null hypothesis and standard deviations for measurements on both types of models**

		**Axis**	**Mean [mm]**	**95% confidence interval**		**P value**	**Std [mm]**	**95% confidence interval**	
**PM**	**for 3-3**	**x**	0.23	−0.24	0.69	0.30	0.46	0.32	0.84
	**for 3-6**	**y**	0.05	−0.09	0.19	0.46	0.14	0.10	0.26
	**for 3**	**z**	0.05	−0.04	0.13	0.28	0.09	0.06	0.16
	**for 3-1**	**mixed**	−0.01	−0.11	0.08	0.75	0.09	0.06	0.17
**RR**	**for 3-3**	**x**	0.12	−0.24	0.49	0.47	0.36	0.25	0.66
	**for 3-6**	**y**	0.04	−0.04	0.12	0.33	0.08	0.06	0.15
	**for 3**	**z**	0.11	−0.03	0.25	0.10	0.14	0.09	0.25
	**for 3-1**	**mixed**	0.05	−0.08	0.18	0.42	0.13	0.09	0.24

This table shows the results of the paired *t*-test. 3–3 means intercanine distance, x-plane, 3–6 means y-plane, 3 means the clinical hight of crown of canine, 3–1 states for mixed plane. The null hypothesis states that there are no differences between the measurements on the plaster models (PM) and RepRap 3D printed copies (RR). And the samples were randomly selected from a normal distribution with mean 0 and unknown variance. The alternative hypothesis is that that the mean is not 0. In the second part of the table, the values of standard deviation for the measurements on the plaster models and RepRap 3D printed copies are depicted.

The precision of the measurements on the plaster models and RepRap 3D printed copies were estimated from the difference of the repeated measurements (Table 1). Let X, Y ~ N (μ,σ^2^) be values from the first and the second independent distance measurements, then Z ~ N (0,2σ^2^) is their difference (*Z=X–Y*).

The equity of the measurements on the plaster models and measurements on the RepRap 3D printed copies were confirmed using the paired *t*-test (Table 2). The mean value W ~ N (0,σ^2^/2) was used for this purpose (*W=(X+Y)/*2).

**Table 2:** **Results of the paired *t*-test of the null hypothesis and results of F-test of the null hypothesis**

		**Axis**	**Mean [mm]**	**95% confidence interval**		**P value**	**Value of the test statistic**	**95% confidence interval**		**P value**
**PM-RR**	**for 3-3**	**x**	−0.17	−0.35	0.01	0.06	1.61	0.40	6.48	0.49
	**for 3-6**	**y**	−0.05	−0.18	0.09	0.47	3.08	0.77	12.4	0.11
	**for 3**	**z**	−0.03	−0.16	0.09	0.55	0.42	0.11	1.71	0.22
	**for 3-1**	**mixed**	0.04	−0.07	0.15	0.45	0.52	0.13	2.08	0.34

This table shows the results of the paired *t*-test. 3–3 means intercanine distance, x-plane, 3–6 means y-plane, 3 means clinical hight of crown of canine, 3–1 states for mixed plane. The null hypothesis states that there are no differences between the measurements on the plaster models (PM) and RepRap 3D printed copies (RR). And the samples were randomly selected from a normal distribution with mean 0 and unknown variance. The alternative hypothesis is that that the mean is not 0. The second part of the table depicts the results of the F-test. The null hypothesis states that the differences of the repeated measurements on the plaster models and the difference of the repeated measurements on the RepRap 3D printed copies are from a normal distributions with the same variance. The alternative hypothesis states that they are from a normal distributions with different variances.

The comparison of precision of the measurements on the plaster models and precision of the measurements on the RepRap 3D printed copies were performed using the F-test of equality of variances [19] (Table 2).

The accuracy of the RepRap 3D printed copies for distance measurements were estimated using standard deviation of differences between the plaster models and the RepRap 3D printed copies (Table 3).

**Table 3:** Sums of standard deviation for the plaster casts and 3D prints and its standard deviation

	**Axis**	**Std (WPM) + Std (WRR)**	**Std (WPM -WRR)**	**WPM-WRR**	**WPM-WCP**
**for 3-3**	**x**	0.58	0.25	−0.19	−0.21
**for 3-6**	**y**	0.16	0.36	−0.05	−0.07
**for 3**	**z**	0.16	0.17	0.11	0.22
**for 3-1**	**mixed**	0.16	0.15	0.03	0.05

This table shows the sums of standard deviations for the plaster models (W_PM_), the RepRap 3D printed copies (W_RR_), and the values of standard deviation for the difference between the plaster models and the RepRap 3D printed copies. 3–3 means intercanine distance, x-plane, 3–6 means y-plane, 3 means clinical hightof crown of canine, 3–1 states for mixed plane. The second part of the table depicts the differences of the representative values of the measurements on the plaster models, the measurements on the RepRap 3D printed copies (W_PM_-W_RR_), the differences of the representative values of the measurements on the plaster models and the measurements on the commercial 3D printed copies (W_PM_-W_CP_).

Data gathered from 3D printed materials obtained from commercial printers couldn’t be statistically evaluated because of insufficient number of data. Table 3 shows differences in measurements made by comparing plaster cast, RepRap prints and commercial prints.

All the tests were performed on the significance level *p*=0.05 , separately for 3–3 (intercanine distance, x-plane), 3–6 (distance between tip of canine – cusp of mesiobuccal tip of the first molar, y-plane), and mixed distances. All the statistical calculations were performed using the Matlab Statistical Toolbox [20].

### Comparison study

**Figure 3:**
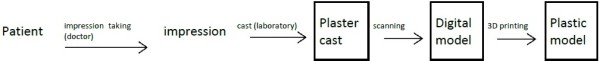
Scheme of the process of formation of plaster casts, digital model and 3D printed model from impression taking to utilization of the digital data as template for 3D printer.

**Figure 4:**
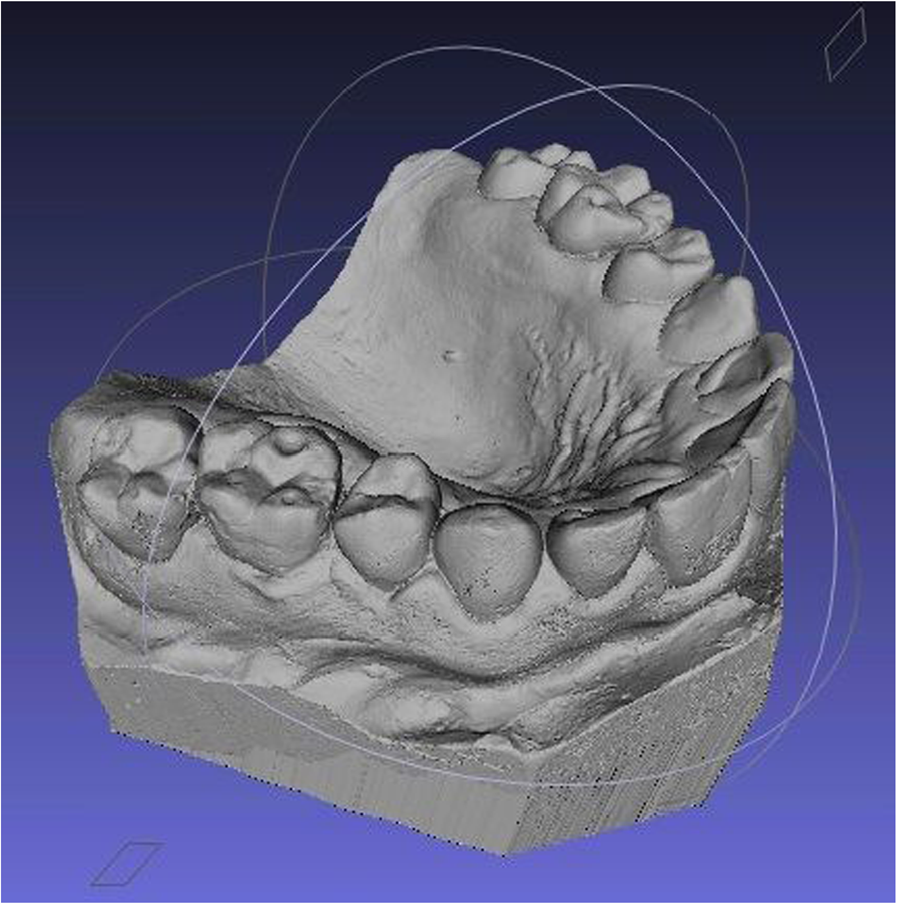
Computer digital model.

The process of formation of plaster cast, digital model and 3D printed model are schematically shown in Figure 3. Common process of impression taking takes part in dental practice and impression is manufactured in dental lab on plaster casts, which is later used as a model scanned by various scanners and data are kept in digital form on mass storage devices (Figure 4). The 3D Model can then be reconstructed from the stored digital data as and when required.

Table 4 shows the comparison study of plaster casts, digital models and 3D printed copies based on various criteria.

**Table 4:** The table shows the comparison study of plaster casts, digital models and 3D printed copies based on the various criteria

		**Plaster model**	**Digital model**	**Printed model (RepRap)**	**Printed model (ProJet D3000)**	**Digital model + Printed model (RepRap)**
**Prices**	**Price of one model**	order of 1 EUR	0 EUR	order of 1 EUR	order of 10 EUR	order of 1 EUR
	**Price of equipment**	-	order of 10 000 EUR	order of 10 EUR	order of 10 000 EUR	order of 10 EUR
	**Price of material**	-	-	order of 10 EUR/kg	order of 10 EUR/kg	order of 10 EUR/kg
**Properties**	**Color**	any color	any color	blue	pale yellow	blue
	**Transparency**	no	yes/no	no	yes	no
	**Weight model**	heavy	none	light	light	light
		70 g		20 g	40 g	
	**Liable to damage**	high	none	low	low	low
	**Stable material**	yes	-	yes	yes	yes
	**Heat resistance**	high	-	low	low	low
	**Harmless material**	yes	-	yes	yes	yes
**Other**	**Reproducibility**	no	yes	no	no	yes
	**Pose storage problem**	yes	no	yes	yes	no
	**Access and transfer problem**	yes	no	yes	yes	no
	**Retrieval problem**	yes	no	yes	yes	no

## Results

The precision of the distance measurements on the plaster models and on the RepRap 3D printed copies are the same. This was confirmed by the estimated characteristics including confidence intervals (Table 1).

The distance measurements on the plaster models and on the RepRap 3D printed copies were equivalent. There was no significant difference between them (evaluated with paired *t* test, Table 2).

The accuracy of the RepRap 3D printed copies for distance measurements (with respect to the plaster models) was estimated using standard deviation of differences between the plaster models and the RepRap 3D printed copies. The standard deviation values did not significantly differ (or were even smaller) than the sum of the standard deviation values of the plaster models and the RepRap 3D printed copies (Table 3). All standard deviation values were less than 0.5 mm.

These conclusions are valid for all the distances measured.

## Discussion

This study proposes a novel method for reconstruction of digital data using 3D printed dental study models. The advantages and disadvantages of conventionally used plaster casts, digitally scanned models and 3D printed copies are also presented here.

Although advantages of 3D printing have been mentioned by numerous authors [7,21] here we present a summary of these attributes in comparison with the traditional plaster casts (Table 4):

Advantages: low weight, low probability of fracturing and damage to most materials, durability, high resistance to abrasion, transportability and most importantly the possibility of sharing digital data. Furthermore there is the possibility of producing new 3D models on demand which in turn will eliminate the burden of storage problem. There is also the potential to print using environmentally friendly materials.

Disadvantages: need for a trained technician, legalities of sharing patients’ data, low heat resistance of material used, possible loss of the digital data due to technical errors.

One of the main issues surrounding 3D printing is its high cost. However to circumvent the cost issue we can use an open source printer such as RepRap which has a low overall cost per print (unlike commercial printer ProJetHD3000, Table 4). Although the costs related with the printer usage are much lower than plaster casts, the printer needs the presence of a qualified individual to avoid complications resulting in errors in the process of 3D printing. However this is no different to the need for a qualified lab assistance required for production of plaster casts.

**Figure 5:**
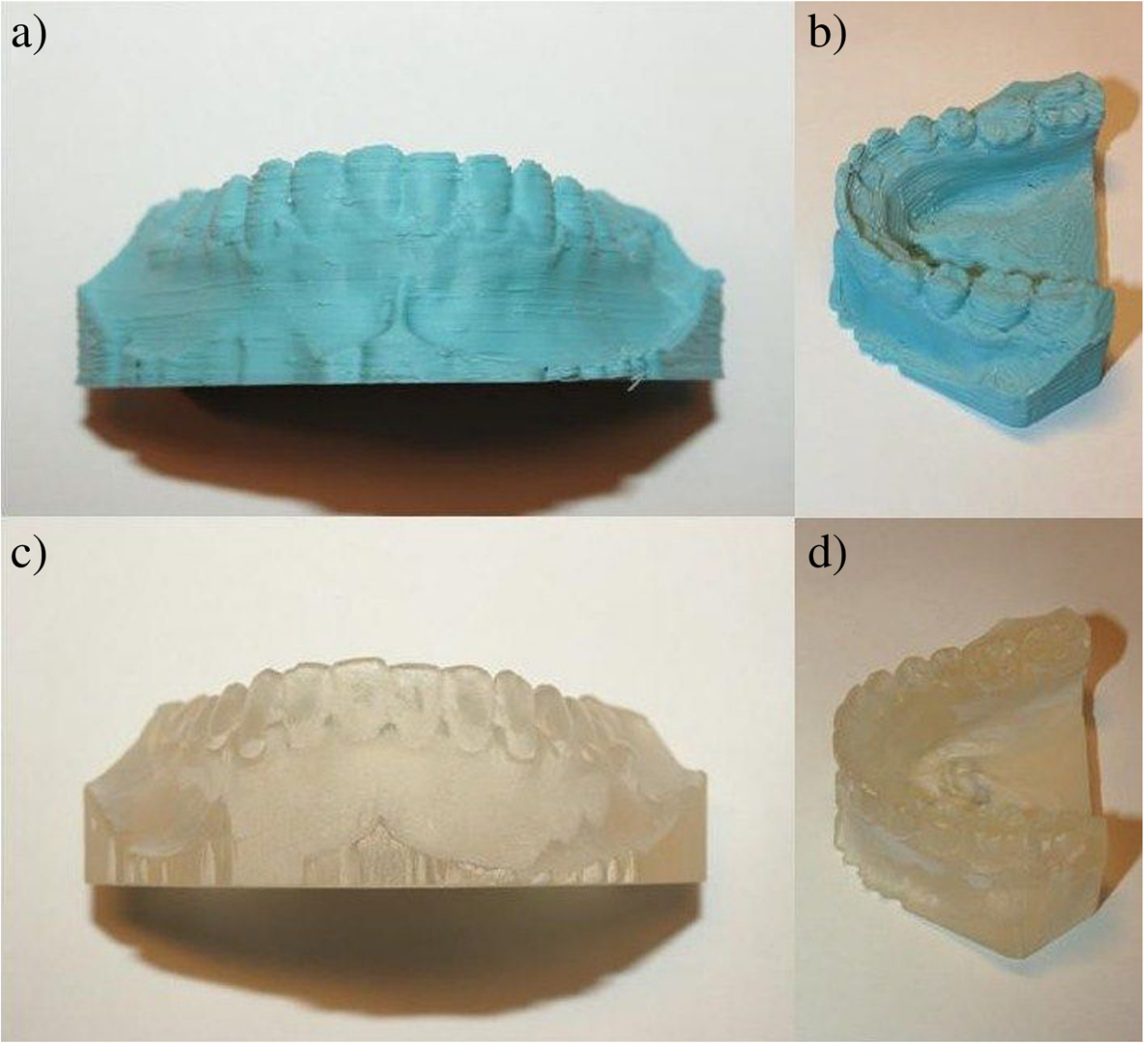
**Difference between RepRap printed and commercial printed plaster casts. a**) RepRap print – bucal view, **b**) RepRap print – semi occlusal view, **c**) commercial print – bucal view, **d**) commercial print – semi occlusal view.

The necessary condition which needs to be met in order to use any 3D printed copies of the traditional plaster casts in dentistry is its accuracy and precision. Although it is evident, that casts printed on commercial ProJet D3000 printer are of higher quality (Figure 5), we did not encounter any difference in the two for clinical purposes. Statistical analysis using standard tests confirmed the accuracy and precision of the measurements on the RepRap 3D printed copies. Here we can conclude that 3D printing can be used as a reliable method of reversing stored 3D digital data of the previously scanned plaster casts. This lack of significant difference is an important notion which could potentially sustain clinical advantages such as creation of digital libraries and the possibility of being able to obtain a physical plaster cast when required.

**Figure 6:**
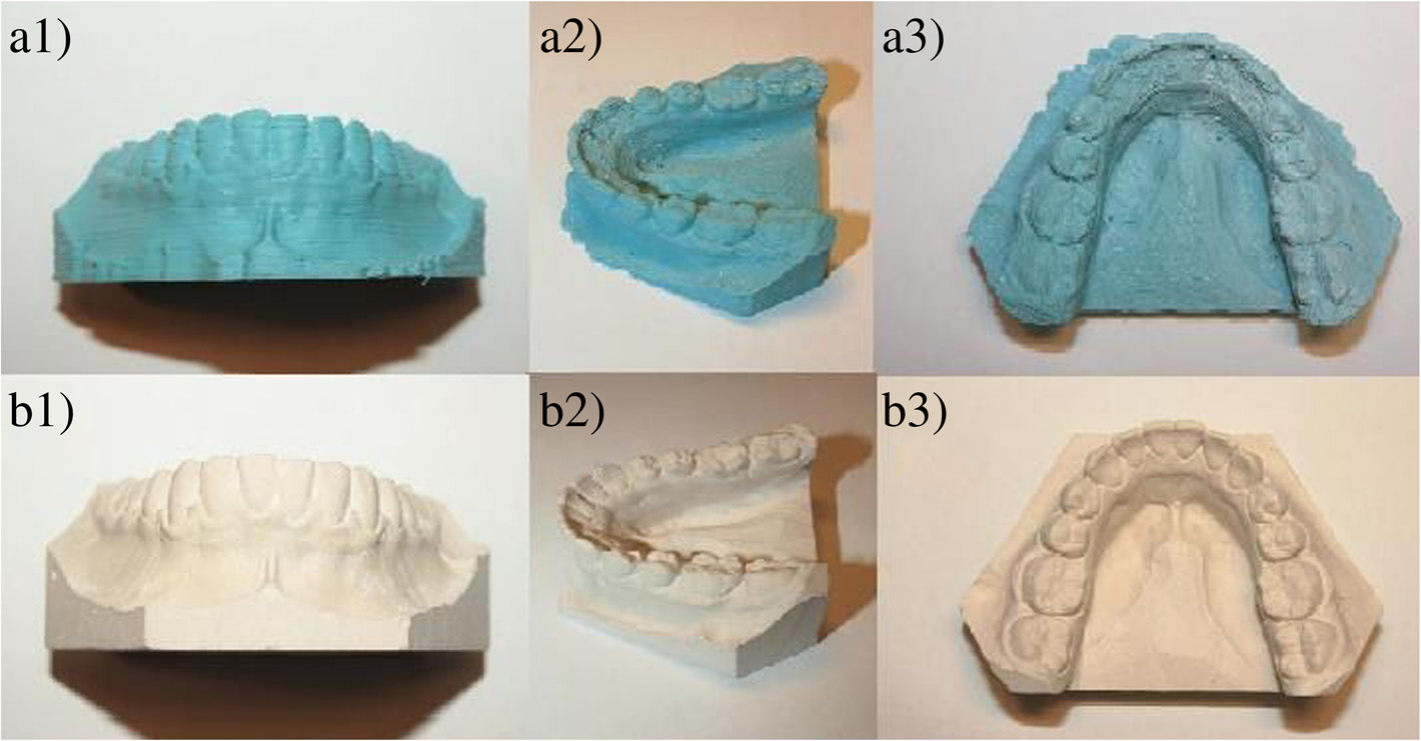
**Comparison of traditional plaster cast from bucal (a1), semi occlusal (a2) and occlusal (a3) point of view with RepRap print from bucal (b1).** Semi occlusal (**b2**) and occlusal (**b3**) point of view.

In comparison, event though ProJet has a higher resolution and accuracy, RepRap on the hand benefits from a low price and faster printing as well as the possibility of using various materials with different properties and a small size of the printer (Figure 6). Furthermore the quality of print in RepRap printer can be greatly improved with little investment.

Keeping patients’ data in digital form can help solve the storage problem, as all of the study models must be kept for certain period of time, which requires a large storage space. On the other hand when the 3D printed plaster casts are no longer required, they can be recycled and reused. This is an environmentally sensible choice as opposed to the traditional gypsum cats. Digitalization of the data also offers a solution for retrieval and transfer of the patients’ data as and when required by the team of various physicians responsible in the care of the patient. However from the legal point of view one must be vigilant when sharing patients’ data as there are potential confidentiality and privacy issues that could be prone to misuse. It is believed that 3D printed objects could play an increasingly important role in many fields of research. Further investigations are warranted in this field and there is a need to focus on simplifying the process of scanning and 3D printing. There is also a scope for reconstructing data for 3D printers directly from other source data such as ortopantomograph, CT scans or direct scanning of patients’ mouth to eliminate the intermediary step of manufacturing traditional plaster casts.

## Conclusions

1. It is possible to construct digital data to obtain 3D printed models.

2. The distance and the precision of the measurements on the plaster models and on the RepRap 3D printed copies are equivalent.

3. RepRap 3D printed model can replace the process of plaster making.

4. The RepRap 3D printer has the advantage of being the cheaper and faster option when compared to ProJe**t** D3000 and it can potentially replace traditional plaster casts.

## Abbreviations

FDM: Fusion deposition modeling; SLS: Selective laser sintering; SLA: Stereolitography; CT: Computed tomography; 3D: Three dimensional; PM: Plaster models; RR: RepRap printed copy.

## Competing interests

There are no relationships or financial support which may pose conflict of interest.

## Authors’ contribution

All authors have contributed significantly and all of the authors are in agreement with the content of the manuscript. MK measured the linear distances, took part in choosing the study group and in scanning plaster casts as well as in data evaluation, TD supervised choosing of patients, took part in data evaluation, LG evaluated data statistically, took part in measuring objects, PD scanned plaster casts, took photos of studied objects and contributed to data analysis, AP supervised 3D printing and comparing of 3D prints, JP printed 3D prints and contributed in technical specification of 3D printers, HE took part in data evaluation and scanning, SK provided language correction.
